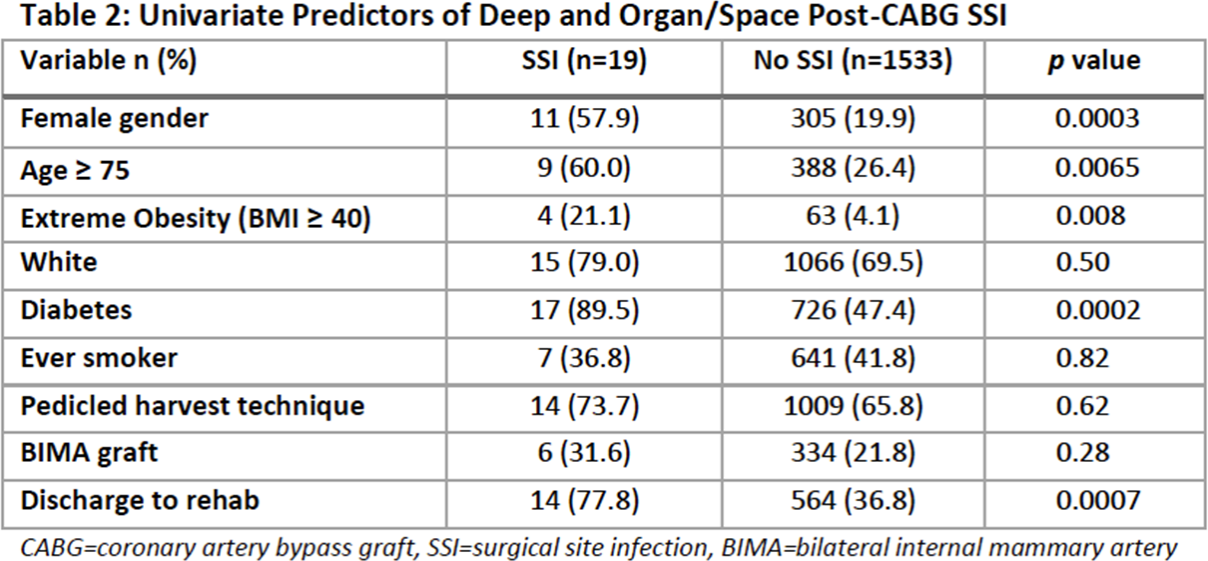# Incidence and Risk Factors for Surgical Site Infection Following Coronary Artery Bypass Graft Procedures

**DOI:** 10.1017/ash.2021.152

**Published:** 2021-07-29

**Authors:** Polly van den Berg, Sharon Wright, Baevin Feeser

## Abstract

**Background:** Deep and organ-space surgical site infections (SSIs) are serious complications of coronary artery bypass graft (CABG) procedures. It is unclear whether the use of bilateral versus single internal mammary artery (BIMA vs SIMA) and surgical approach to internal mammary artery (IMA) harvest (pedicled vs skeletonized) are independent risk factors for SSI. The use of BIMA grafting redirects blood flow away from the sternum to the heart and may increase SSI risk due to lower tissue perfusion. A skeletonized approach to graft harvest, wherein the IMA is dissected free of surrounding tissue to preserve collateral sternal blood flow, may decrease SSI risk as compared to a pedicled approach in which the IMA is mobilized within a tissue pedicle. We describe the incidence and potential risk factors for post-CABG SSI in an academic tertiary-care center performing ~500 IMA procedures annually. **Methods:** Data were abstracted on adult patients who underwent a CABG procedure using at least 1 IMA graft between July 2017 and June 2020. Additional data on potential risk factors for SSI were obtained electronically from hospital data marts and the Division of Cardiac Surgery database, including demographics, comorbidities, number of arterial grafts, surgical approach, surgeon, and discharge location. Using standard NHSN definitions, infection control practitioners identified post-CABG deep and organ-space SSIs. Patient and procedure characteristics were evaluated as potential risk factors for deep and organ-space SSI using the Fisher exact test. **Results:** Of 1,591 CABG procedures performed during the study period, 1,244 (78.2%) were performed using a SIMA technique and 347 (21.8%) were performed using a BIMA technique. The overall post-CABG SSI incidence was 1.2 per 100 procedures, with 1.0 SSIs per 100 SIMA procedures and 1.7 SSIs per 100 BIMA procedures. Table 1 demonstrates an increase over time in proportion of CABG procedures performed using SIMA and skeletonized IMA grafts. We also observed a decrease in overall SSI incidence over this period. See Table 2 for univariate predictors of post-CABG SSI. **Conclusions:** Female sex, BMI ≥40, age ≥75 years, diabetes, and discharge to a rehabilitation setting were associated with development of post-CABG SSI. Although the overall incidence of deep and organ-space SSI in our cohort was very low, making it difficult to draw conclusions about potentially modifiable risk factors, an increase in the use of SIMA and skeletonized grafts appears to be accompanied by a decrease in SSI incidence. More data from our institution and others are needed to determine the significance of this trend.

**Funding:** No

**Disclosures:** None

**Table 1.**
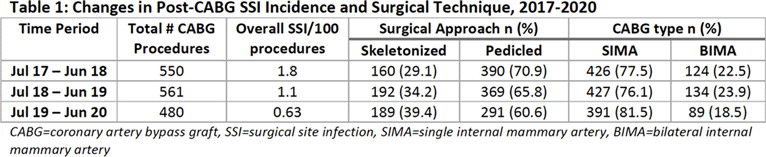

**Table 2.**